# Optimization of centrifugal fan for oolong tea shaking machine based on Kriging model and multi-objective particle swarm algorithm

**DOI:** 10.1038/s41598-025-32474-0

**Published:** 2025-12-19

**Authors:** Chengzhi Ruan, Yiwei Chen, Luxuan Zheng, Bo Guo, Jiayou Chen, Jun Yang, Siling Chen

**Affiliations:** 1 Key Laboratory of Agricultural Machinery Intelligent Control and Manufacturing of Fujian ProvinceUniversity, Wuyi University, Wuyishan, 354300 China; 2 Fujian Key Laboratory of Big Data Application and Intellectualization for Tea Industry, Wuyi University, Wuyishan, 354300 China; 3 School of Intelligent Manufacturing Engineering, Liming Vocational University, Quanzhou, 362000 China; 4 School of Mechanical and Electrical Engineering, Wuyi University, 358 Baihua Road, Wuyishan, 354300 China

**Keywords:** Shaking machine, Centrifugal fan, Agent model, Multi-objective optimization, Flow field analysis, Energy science and technology, Engineering

## Abstract

This study optimized the air volume and efficiency of the centrifugal fan in the shaking machine for oolong tea, aiming to enhance its aerodynamic performance and efficiency. Fitting blade profiles by constraining control points of a third-order Bézier curve through key geometric parameters of the blade, and forty-five sample points were generated in the design space by combining Computational Fluid Dynamics (CFD) simulation and Optimal Latin Hypercube Sampling (OLHS). The Multi-Objective Particle Swarm Optimization (MOPSO) algorithm was used to obtain the optimal parameter combination. Flow field analysis showed that after optimization, the recirculation phenomenon in the flow channel between the blades was improved; the strong turbulent kinetic energy at the inlet and outlet of the fan and the leading edge of the impeller tongue was effectively suppressed, the internal flow field was more stable, and the efficiency was improved. the air volume increased by 176.19 m³/h. These results demonstrate the significance of the proposed blade optimization in the aerodynamic performance of the shaking machine. This study addresses the research gap in the multi-parameter collaborative optimization for centrifugal fans used in oolong tea processing equipment, which traditionally relies on single-parameter empirical designs.

## Introduction

As the first of the world’s three most aromatic teas, oolong tea has become the international business card of Chinese tea culture with its unique aesthetics of “green leaves with red edges” and excellent quality of “rocky bones and floral aroma”^1^. In UNESCO’s list of non-heritage of mankind, Fujian Wuyi Rock Tea production technology as the core representative, showing the world the essence of the wisdom of the Oriental tea. Fujian tea with its irreplaceable quality advantages, in the world tea industry map to establish a pivotal position. Especially Wuyi rock tea in the “king of tea” Dahongpao, since the nineteenth century through the Maritime Silk Road to Europe, will be “Wuyi tea flavor champion of the world” reputation. Oolong tea production process has a long history, the production process includes picking, withering, shaking, killing, kneading and drying processes. Among them, the shaking process makes the tea green leaves constantly collided and rubbed against each other by shaking, so that the edge of the leaves is gradually broken, and after fermentation and oxidation, “green leaves with red edges” is produced, which is the key process of making oolong tea^2^, and the quality and taste of the finished product mainly depend on the effective treatment in the process of shaking.

Thanks to the rapid development of the tea industry, tea processing equipment has begun to rise. The traditional oolong tea processing methods generally use manual shaking, but manual shaking exists shaking uneven strength, low efficiency and high cost, not conducive to the tea green water and flavor formation, and seriously affect the production and quality of oolong tea^3^. The tea shaking machine is developed from the traditional manual shaking mode of oolong tea. It is a key device in oolong tea production that enables the tea leaves to form “green leaves with red edges” through collision and friction, and directly affects the quality and taste of the finished product. With the introduction of oolong tea shaking machine, to make up for the shortcomings of manual shaking also provides help for the scale production of tea, so that the production of tea has been significantly improved^4^. The centrifugal fan, as the core component of the shaking machine, sends the hot air into the barrel by rotating and heating with the charcoal stove. This not only enhances the respiration of the tea leaves, but also provides oxygen for the metabolic reactions to promote the transformation of chemical substances within the tea leaves, further accelerating the water withering and the formation of a unique aroma and flavor. However, the existing shaking machine design relies on manual experience, there are insufficient fan air volume, low efficiency and other problems, can not meet the requirements of oolong tea shaking process.

At present, scholars around the world have done a lot of research on the performance optimization of centrifugal fans, mainly focusing on the inlet and outlet mounting angles of the blades of centrifugal machines^5^, the shape of the blades^6^, the inner and outer diameters of the blades^7^ and the number of blades^8^. Tang Rui, Cheng Fan et al.^9^ utilized the support vector regression surrogate model (SVR) to handle multi-response objectives, and adopted the optimized hyperparameters of SVR and the narrowed search range of genetic algorithm to enhance control performance. Meng et al.^10^ investigated the geometrical parameters of the worm casing by using a high-precision Kriging surrogate model in combination with The Non-dominated Sorting Genetic Algorithm Ⅱ (NSGA-Ⅱ) to improve the overall performance of the centrifugal fan. Darvish et al.^11^ investigated the effect of blade angle on the aerodynamic performance of centrifugal fan and the performance of the fan with a high accuracy. The results of the study on the effect of outlet angle on the aerodynamic performance of centrifugal fan show that increasing the outlet angle appropriately can weaken the flow separation in the impeller channel. Ding Tao et al.^12^ constructed the response relationship between single-factor variables and optimization objectives for axial fan collector and carried out optimization design, after numerical verification, it was found that the optimized collector increased the positive vortex distribution in the impeller, which was conducive to the work done by the impeller. Lee et al.^13^ investigated the effect of different fan blade rib shapes on the performance, and found that the fan type with forward curved fan ribs had the best performance. Song et al.^14^ proposed a multidisciplinary robust optimization method to effectively reduce the maximum blade stress by introducing blade curvature constraints and Polynomial Chaos Kriging model while maintaining similar aerodynamic performance improvement. Ding et al.^15^ obtained the optimal tilting blade design parameters by designing the stacking line of tilting and swept-back blades and investigated it using numerical simulation and orthogonal experiments, and the numerical simulation results show that the aerodynamic performance of the wind turbine is improved. Cui Wenhao et al.^16^ used radial basis neural network combined with genetic algorithm to optimize the design of fan impeller, and the wind pressure, static pressure efficiency and full pressure of the fan were improved after optimization. The aforementioned research on centrifugal fan blade design is of significant importance. However, existing optimization efforts primarily focus on single structural parameters, such as blade inlet/outlet angles, blade shape, impeller diameters, and the number of blades, lacking comprehensive multi-parameter combination optimization and sensitivity analysis. Most of the oolong tea shaking machines on the market have the problems of insufficient airflow and low efficiency in the centrifugal fan, which limits the improvement of oolong tea quality, but few scholars have conducted research and analysis on the optimization of the centrifugal fan in the oolong tea shaking machine. To further fill these gaps and solve practical problems, the main objective of this study is to improve the airflow rate and overall efficiency of the fan through a multi-parameter optimization framework.

In recent years, the integration of the Kriging model with the Multi-Objective Particle Swarm Algorithm (MOPSO) has shown significant potential in the field of complex engineering optimization. The Kriging model, as a spatial interpolation method based on Gaussian processes, has been widely used in the fields of aerospace structural design, environmental resource scheduling, and manufacturing process optimization, etc., because of its ability to quantify the prediction uncertainty and efficiently fit high-dimensional nonlinear response surfaces. process optimization. For example, in aircraft aerodynamic shape optimization, Kriging significantly reduces the computational cost of CFD simulation by constructing proxy models. MOPSO, by virtue of its intelligent group search mechanism, exhibits excellent balance between global exploration and local exploitation when solving multi-objective cooperative optimization problems.

In order to solve the problem, this paper firstly adopts the third-order Bezier curve to fit the equal-thickness blade; subsequently, through OLHS design method, the key parameters of the impeller, such as blade inlet mounting angle, blade outlet mounting angle, blade inlet diameter, blade outlet diameter, and blade center angle, are uniformly sampled to generate sample points. After numerical simulation of the sample points based on CFD, different proxy models are constructed to carry out error analysis. Combined with the constructed Kriging model, this paper further implements the global sensitivity analysis of the design variables to quantitatively assess the degree of influence of each variable on the aerodynamic performance of the wind turbine. The blade design variables are optimized by MOPSO, and the highest performance centrifugal fan impeller design scheme is finally obtained. By comparing and analyzing the flow field distribution characteristics in the impeller of the centrifugal fan before and after optimization, the intrinsic mechanism of improving the aerodynamic performance of the centrifugal fan of the oolong tea shaking machine was revealed, and the efficiency improvement effect was verified. The results of this study are of great practical significance for the improvement of tea processing and quality control, and also provide a theoretical basis and technical support for the in-depth optimization of the design of the centrifugal fan of the oolong tea shaking machine.

## Numerical models and methods

### Working principle of shaking machine

**Fig. 1:**
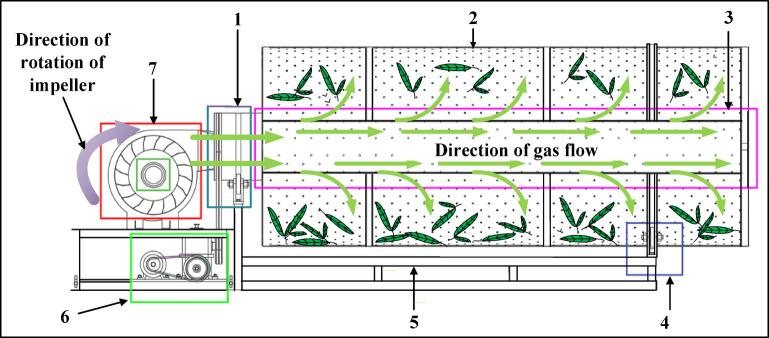
Schematic diagram of shaking machine structure. (1) Roller assembly; (2) rotor roller; (3) internal ventilation pipe; (4) drive assembly; (5) base bracket; (6) drive motor; (7) centrifugal fan.

The structure of the shaking machine is mainly composed of roller assemblies, rotary leaf roller, internal ventilation pipe, centrifugal fan, drive motor, etc. The structure is shown in Fig. 1. The total length of the shaking cylinder is 3.0 m, the diameter is 1.1 m, and the volume of the shaking machine is about 2.85 m^3^. The shaking cylinder is fixed by the shaking cylinder reinforcement, and the wall of the cylinder is designed with a flap of the tea door, which is easy to operate. Through the rotation of the rotary leaf roller, it drives the edge of the tea leaves to collide with each other, and the edge of the leaves is broken, which prompts some components in the tea leaves to change slowly in the air. At the same time, the inlet of the air duct in the shaking cylinder is connected with the outlet of the centrifugal fan, and the inlet of the centrifugal fan is connected with the charcoal stove, which can provide wind and heat for the shaking process to meet the demand of shaking process.

### Geometry of centrifugal fans

**Fig. 2:**
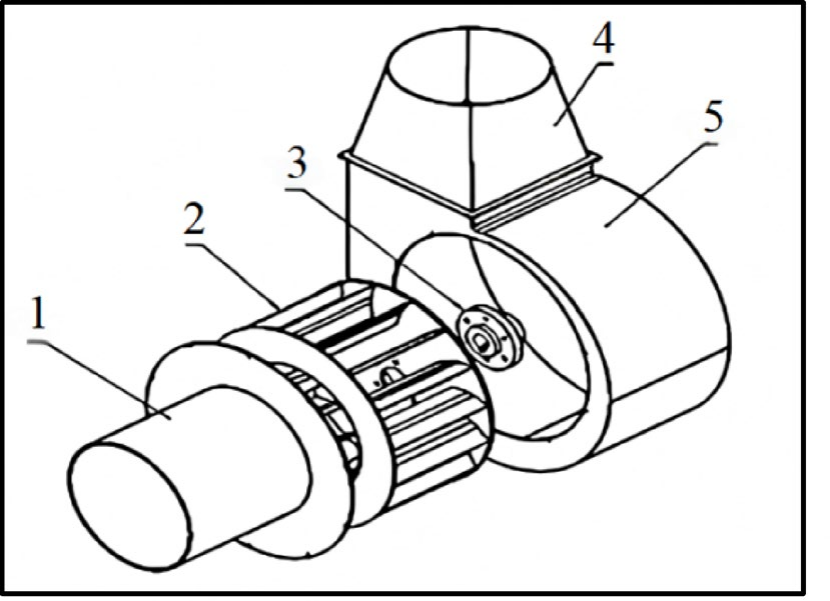
Centrifugal fan structure.1. Air inlet; 2. Impeller; 3. Flange; 4. Air outlet; 5. Worm casing.

This study takes the centrifugal fan in the tea drum type shaking machine as the research object, and the structure is shown in Fig. 2. The fan is divided into impeller structure and worm shell structure, the material is carbon steel. The impeller of centrifugal fan is fixed on the shaft through flange connection, and the shaft is mounted in the rolling bearing on the machine base and connected with the motor, in which the impeller as a rotating part is the key structure that affects the aerodynamic performance of the fan. impeller parameters are shown Table 1.

**Table 1:** Main parameters of impeller structure.

Parameter	Size
Inlet mounting angle,$\:{\:\beta\:}_{1}$/(°)	72.50
Outlet mounting angle,$\:{\:\beta\:}_{2}$/(°)	140.00
Blade inlet diameter,$\:{\:L}_{1}$/mm	45.00
Blade outlet diameter,$\:\:{L}_{2}$/mm	32.50
Blade center angle,$\:\:{\gamma\:}_{1}$/(°)	12.50
Number of blades, $\:Z$	16.00
Impeller outer diameter, $\:{D}_{1}$/mm	400.00
Impeller inner diameter, $\:{D}_{2}$/mm	260.00

### Meshing and irrelevance test for centrifugal fans

**Fig. 3:**
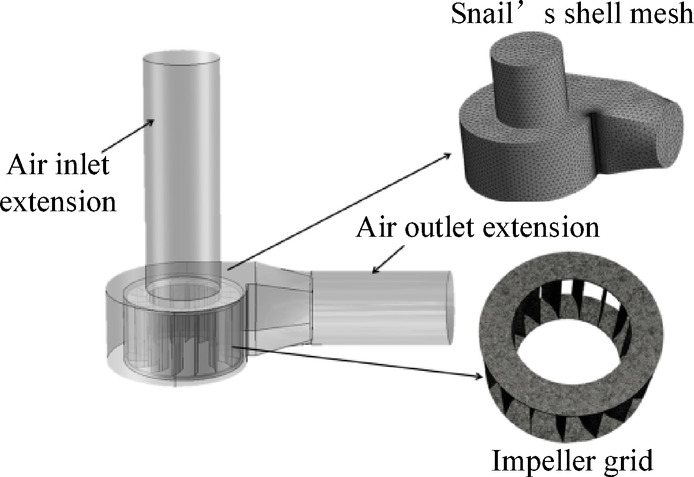
Numerical simulation of centrifugal fan.

The commercial software SOLID WORKS (Dassault Systèmes, version 2022; Vélizy-Villacoublay, France, https://www.solidworks.com/) is used to model the fan in three dimensions, dividing the whole into worm shell domain and impeller domain. In order to ensure the convergence and accuracy of the model created, the cross-sections of the outlet and inlet of the centrifugal fan were extended appropriately^17^. The meshing is performed using ICEM CFD (Ansys Inc., version 2022 R1; Canonsburg, PA, USA, https://www.ansys.com/), and an unstructured mesh is chosen to accommodate complex geometrical features, whose refinement characteristics can accurately capture the details of the flow field and the boundary layer structure. The near-wall surface and the leading and trailing edges of the blades are encrypted to ensure that the y^+^ value of the mesh is in the range of 30 ~ 100 to meet the requirements of the turbulence model^18^. The geometric model of the computational domain and each part of the mesh are shown in Fig. 3.

**Fig. 4:**
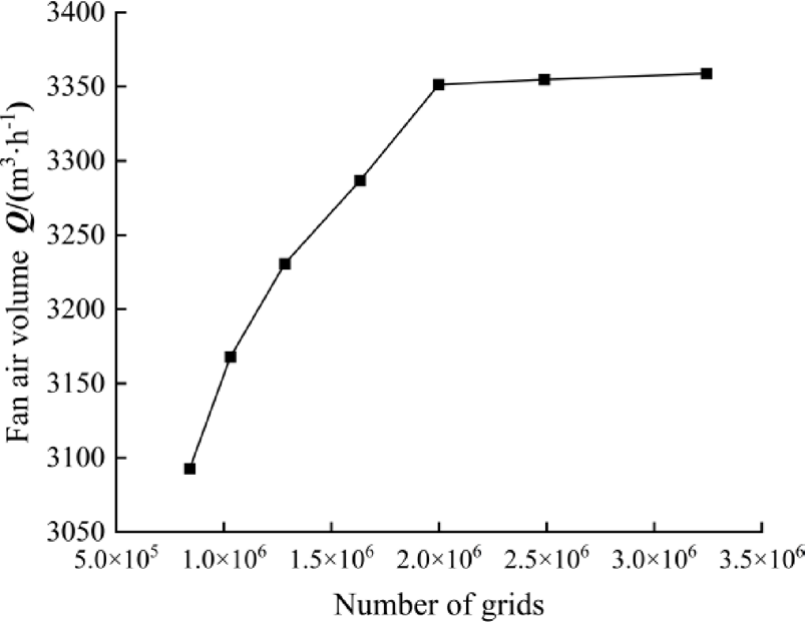
Grid independence verification.

The mesh-independence verification of the original turbine was carried out with the turbine airflow Q as the verification target, and the results are shown in Fig. 4. When the overall number of meshes exceeds 2 million, the airflow basically no longer changes with the increase of the number of meshes, and the airflow fluctuates within the range of 0.15%. Therefore, in order to ensure the calculation accuracy and improve the calculation efficiency, the subsequent studies all adopt the number of 2.4 million grid division scheme.

### Calculation method for centrifugal fans

Numerical simulation calculations of a prototype centrifugal fan modeled in multiple reference systems are performed using Ansys Fluent 2022R1 solver. For the constant flow field calculation, the governing equations are the three-dimensional averaged Navier-Stokes equations as shown in Eq. (1)

1 $$\:\left\{\begin{array}{l}\frac{\partial\:\overline{{u}_{i}}}{\partial\:{x}_{i}}=0\\\:\overline{{u}_{j}}\frac{\partial\:\overline{{u}_{i}}}{\partial\:{x}_{j}}=-\frac{1}{\rho\:}\frac{\partial\:\overline{p}}{\partial\:{x}_{i}}+v\frac{{\partial\:}^{2}\overline{{u}_{i}}}{\partial\:{x}_{j}\partial\:{x}_{j}}\end{array}\right.$$

where $\:\overline{{u}_{i}}\:$is the time-averaged velocity component in the ith direction, m/s; $\:{x}_{i}$ is the ith space coordinate; $\:\overline{{u}_{j}}$ is the time-averaged velocity component in the jth direction, m/s; $\:{x}_{j}$ is the jth space coordinate; i is the direction of x, y, and z; $\:\overline{p}$ is the time-averaged pressure, Pa; $\:v$ is the kinematic viscosity, m^2^ /s.

The turbulence model is the Standard k-epsilon model^19^, and the standard wall function is used for the near-wall treatment. The rotational domain of the impeller region is defined as a kinematic reference system, the rotational speed is set to 1420 r/min, and the no-slip boundary condition is used for the boundary conditions of the wall^20^. The impeller wall surface is defined as a moving rotating wall surface relative to the neighboring unit region, i.e., the interface between the impeller zone rotation domain and the worm shell domain is intersected through the INTERFACE, and the pressure and velocity coupling is performed using the Semi-Implicit Method for Pressure-Linked Equations solver^21^. In the numerical discretization of the flow control equations, the momentum equation, energy equation and turbulence dissipation equation of the control equations are used in Second Order Upwind discrete format and the pressure term is used in PRESTO! format^22^. The inlet boundary condition is given as the total pressure of 0 Pa, and the outlet boundary conditions are all given as the static pressure of 0 Pa, where both pressures are 0 Pa gauge pressure relative to the standard atmospheric pressure, so as to simulate the actual working conditions of the fan sucking air from the environment and exhausting it into the atmosphere. The convergence residuals of K, epsilon and viscous terms are set to be 10^− 5^, and the number of iterations is 1000.

### Fan performance test

**Fig. 5:**
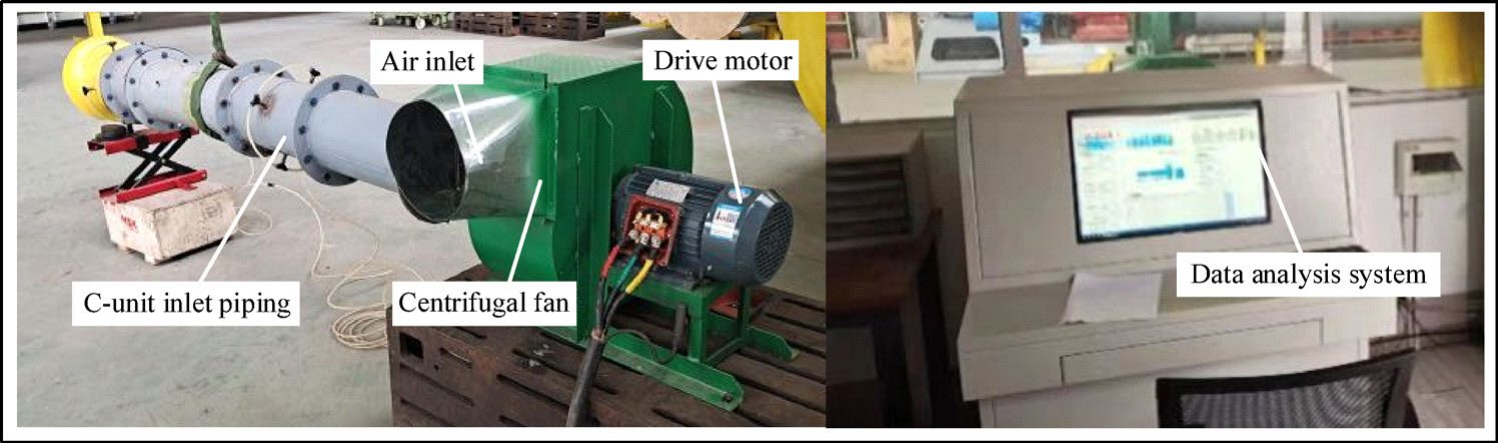
Experimental test rig platform.

The performance of the centrifugal fan is tested using the C-type experimental setup of the national standard GB/T 1236–2017 “Industrial fan-Performance testing using standardized airways” to test the aerodynamic performance of the fan, and the experimental setup was shown in Fig. 5. The experimental platform is mainly composed of absolute pressure transmitter, pressure transmitter, power synthesizer and tachometer and other instruments. During the experiment, a stable indoor ventilation environment was maintained, and the electric power measurement method combined with a data analysis system was adopted. The test was conducted by fixing the arc-shaped air inlet section to connect the air inlet of the fan performance test equipment with the air inlet pipe, so as to obtain the performance parameters of the fan, such as air volume and efficiency. The test accuracies of different instruments are as follows: the accuracy of the tachometer is ± 1 revolutions per minute, the accuracy of the power synthesizer is ± 2%, and the accuracies of both the absolute pressure transmitter and the pressure transmitter are ± 0.6%.

## Optimized design solutions

### Impeller blade design

**Fig. 6:**
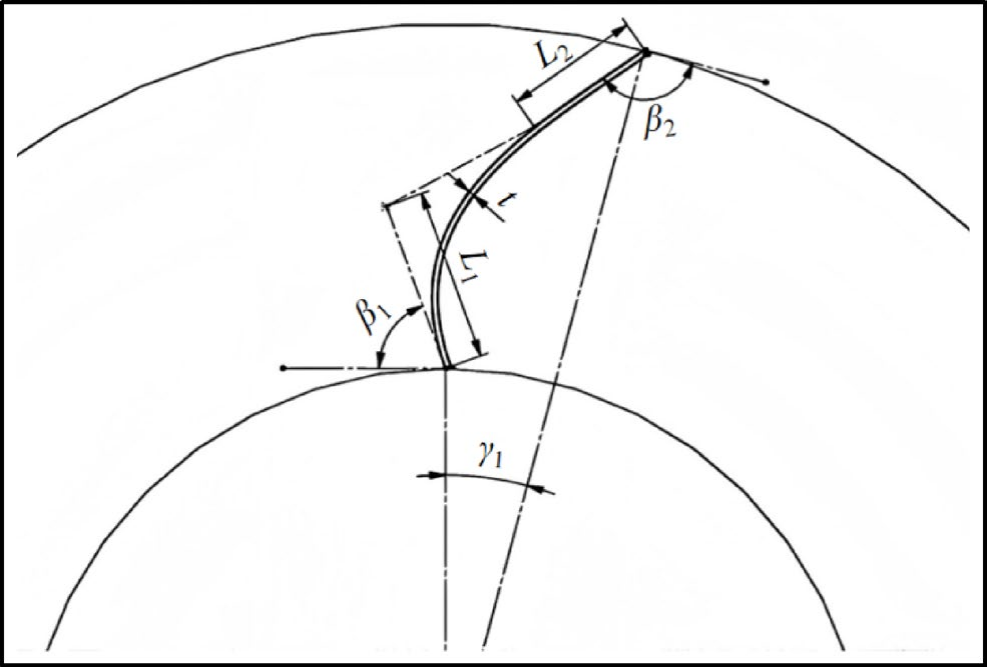
Schematic diagram of blade design parameters.

First of all, the blade optimization design, under the premise of keeping the worm shell unchanged and the blade profile smooth, a third-order Bézier curve was used for geometric parametric fitting of the equal-thickness blade^23^. The specific fitting method is as follows: Five key geometric parameters of the blade—blade inlet mounting angle$\:{\:\beta\:}_{1}$, blade outlet mounting angle$\:{\:\beta\:}_{2}$, blade inlet diameter $\:{L}_{1}$, blade outlet diameter$\:{\:L}_{2}$, blade center angle$\:\:{\gamma\:}_{1}$—were used to impose spatial position and directional constraints on the four control points of the third-order Bézier curve (blade inlet endpoint, two intermediate control points, and outlet endpoint). Through this approach, accurate matching between the curve and the blade profile was achieved, and the fitting schematic diagram is shown in Fig. 6. In this paper, the blade inlet mounting angle *β*_1_, blade outlet mounting angle *β*_2_, blade inlet diameter *L*_1_, blade outlet diameter *L*_2_, blade center angle *γ*_1_ as the independent variable, according to the actual design experience of each design parameter value range: *β*_1_ = 65°~80°, *β*_2_ = 125°~155°, *L*_1_ = 35 ~ 55 mm, *L*_2_ = 20 ~ 45 mm, *γ*_1_ = 9°~16°. Select the fan air volume *Q*, the overall efficiency as the target parameters for multi-objective optimization of centrifugal fans to screen out the best design parameters. The mathematical model of multi-objective optimization is shown in Eq. (2) :

2 $$\:\left\{\begin{array}{l}{max}\,Q({\beta\:}_{1},{\beta\:}_{2},{L}_{1},{L}_{2},{\gamma\:}_{1})\\\:{max}\, \eta\:({\beta\:}_{1},{\beta\:}_{2},{L}_{1},{L}_{2},{\gamma\:}_{1})\\\:s.t.\left\{\begin{array}{l}\begin{array}{c}65\le\:{\beta\:}_{1}\le\:80\\\:125\le\:{\beta\:}_{2}\le\:155\end{array}\\\:35\le\:{L}_{1}\le\:55\\\:20\le\:{L}_{2}\le\:45\\\:9\le\:{\gamma\:}_{1}\le\:16\end{array}\right.\end{array}\right.$$

### Surrogate modeling and sensitivity analysis

**Fig. 7:**
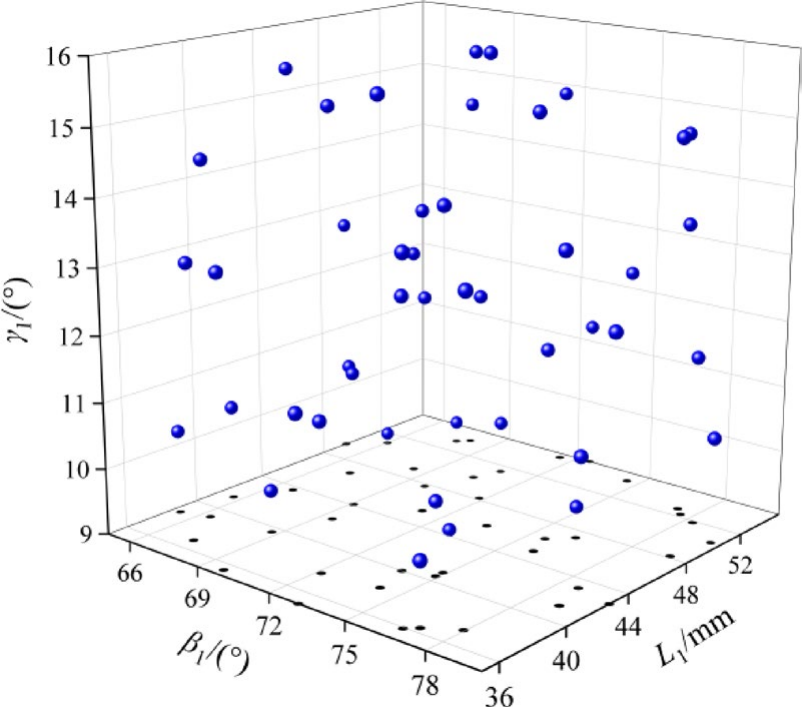
Spatial distribution of samples.

A surrogate model is usually an approximate mathematical model that can be used to describe the relationship between the inputs and outputs of a complex system. Before the surrogate model is established, the sample points should be generated firstly based on the experimental design method, and the distribution of the design variables in the design space has an important impact on the accuracy of the surrogate model. In order to obtain a reasonable spatial distribution of data, this paper adopts OLHS^24^ to generate forty-five groups of samples, compared with the traditional sampling method OLHS has a higher spatial filling capacity, the spatial distribution of the samples is shown in Fig. 7. Taking the blade inlet mounting angle *β*_1_, blade inlet diameter *L*_1_, blade center angle *γ*_1_ as an example, the distribution of sample points in the three-dimensional space and the two-dimensional projection plane can be seen in the set range of independent variables, the spatial distribution of sample points is relatively uniform, which is conducive to the construction of the agent model. CFD numerical simulation of forty-five groups of sample points, using the same calculation method as the original fan, to maximize the fan air volume and fan efficiency as the goal to determine the numerical simulation of the working conditions of each test scenario^25^, the numerical simulation results are shown in Table 2.

**Table 2:** Experimental design scheme and results.

Serial number	β_1_/(°)	β_2_/(°)	L_1_/mm	L_2_/mm	γ_1_/(°)	Q/( m^3^/h)	η/%
1	77.95	144.77	37.27	20.57	13.61	4090.31	34.34
2	69.43	129.09	42.27	41.02	15.20	4115.48	34.95
3	66.70	148.86	43.64	31.93	15.52	4101.61	34.17
4	67.73	128.41	53.18	30.80	9.32	4115.18	34.81
5	66.02	147.50	50.91	34.20	9.16	3973.05	35.21
6	75.91	153.64	45.00	27.39	15.36	4073.93	33.95
7	73.86	154.32	40.00	38.75	12.98	4124.73	35.46
8	76.59	140.00	35.91	44.43	14.09	4096.52	35.28
9	66.36	132.50	39.09	26.82	14.41	4050.16	34.34
10	79.32	137.27	48.18	24.55	15.05	4005.45	33.92
11	72.5	140.68	55.00	35.91	11.23	4030.75	35.12
12	78.64	133.18	43.18	22.27	10.91	4081.32	35.03
13	76.93	143.41	36.36	39.89	9.95	4007.53	35.06
14	78.98	126.36	41.36	36.48	13.93	4121.42	35.15
15	77.61	152.95	52.27	39.32	13.45	4095.31	35.24
16	70.45	129.77	50.00	27.95	15.68	4010.24	34.21
17	67.39	142.05	36.82	40.45	13.14	4106.81	35.53
18	77.27	133.86	52.73	38.18	14.73	4111.13	34.99
19	69.77	150.91	35.45	26.25	13.30	4108.62	34.79
20	76.25	152.27	47.27	37.61	9.48	3988.35	35.23
21	74.55	130.45	54.09	23.41	12.34	4004.46	34.66
22	67.05	134.55	46.82	43.86	10.75	4021.37	34.95
23	74.89	125.68	40.91	22.84	14.25	4018.12	34.21
24	68.41	151.59	49.09	43.30	12.5	4081.68	35.47
25	68.07	136.59	53.64	41.59	14.57	4105.97	35.09
26	72.16	127.73	37.73	42.73	11.23	4030.47	35.18
27	71.14	141.36	45.45	20.00	13.61	4022.81	34.35
28	69.77	155.00	47.73	27.95	12.02	4035.86	35.56
29	72.84	135.91	44.55	32.50	9.00	3993.39	34.89
30	65.68	144.77	41.36	23.98	10.43	4055.55	35.53
31	68.75	150.23	39.55	36.48	9.64	3990.71	34.26
32	80.00	144.09	42.73	33.64	12.82	4129.92	35.46
33	65.34	131.14	38.64	33.07	10.27	4016.50	34.89
34	71.82	125.00	48.18	35.34	12.18	4084.05	35.15
35	75.57	139.32	46.36	45.00	11.86	4064.93	35.53
36	70.80	142.73	51.36	21.14	9.80	4023.25	35.49
37	69.09	127.05	44.09	21.70	11.07	4065.17	34.98
38	73.18	134.55	35.00	29.09	11.70	4080.12	35.30
39	79.66	131.82	50.45	34.77	10.59	4049.32	34.78
40	71.48	146.82	54.55	29.66	14.89	4022.98	34.21
41	78.30	148.18	51.82	25.68	11.55	4022.98	35.42
42	74.20	138.64	38.18	31.36	15.84	4101.26	34.12
43	65.00	137.95	49.55	30.23	12.66	4026.18	35.13
44	73.52	146.14	45.91	42.16	16.00	4111.23	34.66
45	74.89	149.55	40.45	25.11	10.11	4074.46	35.51

**Fig. 8:**
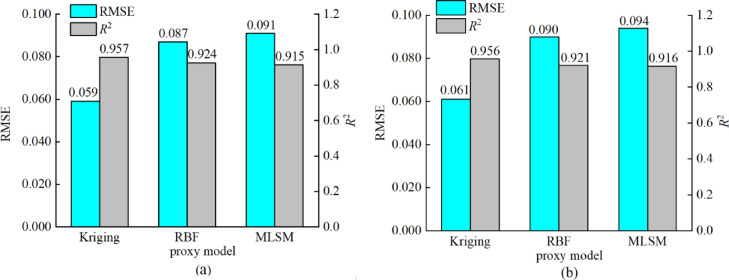
Different proxy model tests: (**a**) Fan airflow proxy model test; (**b**) Fan efficiency proxy model test.

Based on forty-five sets of sample data, a variety of agent models were selected to screen the optimal agent model between the constructed objectives and design variables. In order to test the accuracy of the proxy model, the coefficient of determination (*R*^2^) and the root mean square error (RMSE) are used as evaluation indexes^26^ to analyze the error of the constructed proxy model and screen out the optimal proxy model. Among them, the closer the *R*^2^ value is to 1 and the smaller the RMSE value is, it indicates that the agent model has a higher fitting accuracy. The error accuracy of each model is shown in Fig. 8. The analysis results showed that the Kriging proxy model had the highest accuracy, with *R*^2^ of 0.957 and 0.956 for fan airflow *Q* and overall efficiency$\:\:\eta\:$, respectively, and RMSE of 0.059 and 0.061, respectively, and it can be concluded that the Kriging proxy model can accurately construct the functional relationship between the objective and the optimization variables.

The Kriging model is based on Gaussian process theory. To capture the global linear correlations between design variables and outputs, a first-order linear trend term is selected. It jointly characterizes the complex relationships between variables through the collaboration of the global trend term and local random deviations. Its core advantage lies in the ability to quantify prediction uncertainty, making it suitable for high-dimensional nonlinear engineering problems^27^. The kernel function determines the model’s ability to characterize correlations between sample points; this paper adopts the Gaussian kernel function, which has been widely validated in engineering:

3 $$\:R\left(d\right)=\mathrm{exp}\left(-\frac{1}{2}\sum\limits_{i=1}^{5}{\left(\frac{{d}_{i}}{{\theta\:}_{i}}\right)}^{2}\right)$$

In the formula, $\:{d}_{i}$ the Euclidean distance vector between any two sample points in the design space; $\:{\theta\:}_{i}$ is the anisotropic scaling factor for each design variable, which serves to adjust the sensitivity weights of different variables with respect to the output. After hyperparameter optimization, the values of each $\:{\theta\:}_{i}$ are as follows:$\:{\theta\:}_{1}$ = 0.02$\:{\beta\:}_{1}$, $\:{\theta\:}_{2}$ = 0.025$\:{\beta\:}_{2}$, $\:{\theta\:}_{3}$ = 0.032$\:{L}_{1}$, $\:{\theta\:}_{4}$ = 0.039$\:{L}_{2}$, $\:{\theta\:}_{5}$ = 0.028$\:{\gamma\:}_{1}$.

**Fig. 9:**
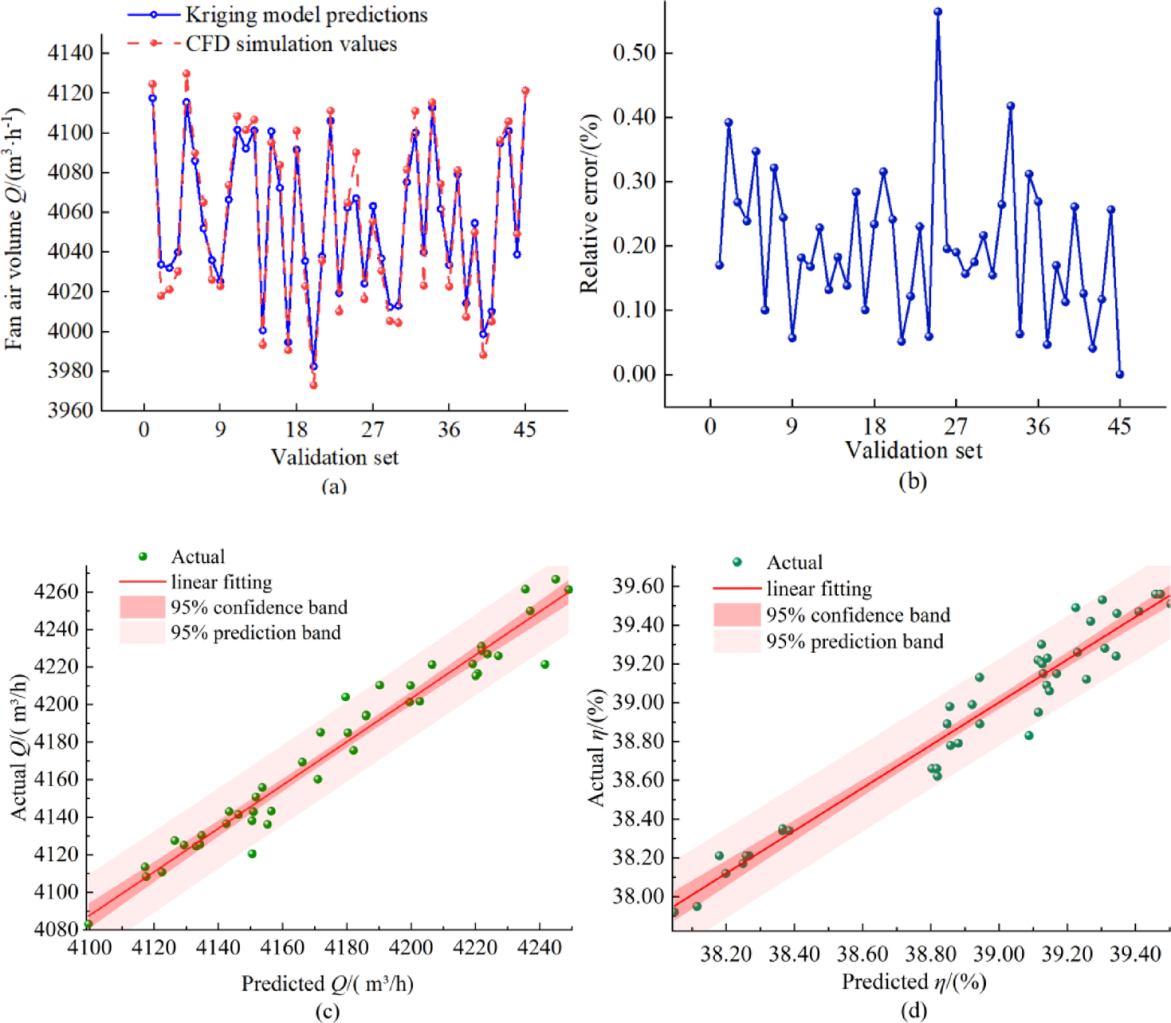
Kriging model prediction results and prediction errors: (**a**) Comparison of Kriging model predictions with CFD simulations; (**b**) Relative errors of Kriging model predictions with CFD simulations; (**c**) Kriging model parity plots for fan airflow; (**d**) Kriging model parity plots for efficiency.

In order to verify the fitting accuracy of the Kriging agent model, the predicted values of the Kriging surrogate model are compared and analyzed with the CFD numerical simulation results in this paper,

The comparison results were shown in Fig. 9a, which indicated that the predicted values of the Kriging model were very close to the CFD simulation values. The relative errors between the predicted values of the Kriging surrogate model and the CFD numerical simulation are shown in Fig. 9b, which show that the relative errors between the predicted values and the simulated values are less than 0.6%. In Figs. 9c and d, the predicted values of airflow and efficiency by the Kriging model are closely distributed on both sides of the ideal fitting line, with no obvious dispersion trend. Meanwhile, via maximum likelihood estimation, the objective function was selected as “maximizing the model’s log-likelihood value”. The maximum number of iterations was set to 300, and the convergence threshold was set to 1 × 10^5^. The log-likelihood value of the model after final optimization is − 2.3. This value was determined by minimizing the leave-one-out cross-validation error. Therefore, it can be considered that the fitting accuracy of the Kriging surrogate model constructed in this paper is high.

Global Sensitivity Analysis (GSA) is an important method used to quantify the extent to which a model’s input variables influence the output variables^28^. Through GSA, the contribution of each input variable to the output variable can be evaluated to identify the variables that have the most significant impact on the output. Among the many global sensitivity analysis methods, the Sobol index method is a representative one. Its core idea is based on variance decomposition, which decomposes the output variance of a model into the contributions of individual input variables and their interaction terms. By calculating the proportion of the variance of a single input variable or set of variables to the total output variance, the Sobol Index method is able to effectively assess the sensitivity of input variables and identify the key parameters that have the greatest impact on the output.

**Fig. 10:**
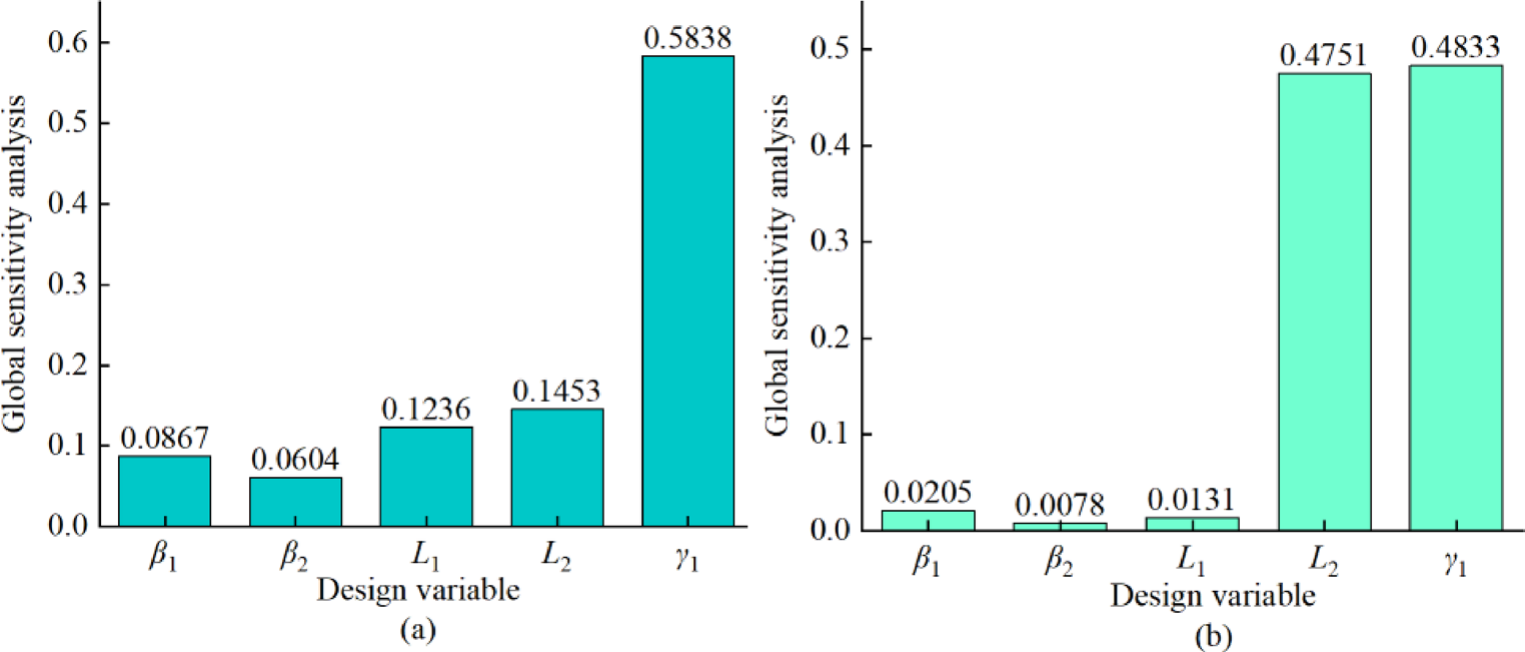
Global sensitivity analysis results: (**a**) Global sensitivity of each design variable to airflow; (**b**) Global sensitivity of each design variable to efficiency.

In this paper, when using the Sobol method to solve the global sensitivity, OLHS is firstly used to extract 1000 sample points within the sample interval, and then the output values of the sample points are predicted according to the trained Kriging agent model, and the global sensitivity analysis is carried out for each design variable^29^, and the sensitivity analysis of the design variables to the target parameters is shown in Fig. 10, it can be seen that the parameter *γ*_1_ has the most significant degree of influence on the fan air volume, and its sensitivity index is 0.5838. The parameters *L*_2_ and *γ*_1_ both show a large degree of influence on the fan efficiency, and their sensitivity indexes are 0.4751 and 0.4833, respectively.

### Analysis of optimization results

The overall optimization workflow, including the automated execution of CFD simulations, the construction and evaluation of the Kriging surrogate model, and the implementation of the Multi-Objective Particle Swarm Optimization (MOPSO) algorithm, was integrated and managed using the Isight process integration and design optimization platform (Dassault Systèmes, version 2022, Vélizy-Villacoublay, France, https://www.3ds.com/zh-hans/products/simulia/isight/). The core algorithms for surrogate modeling and MOPSO were developed and executed using the Python programming language (Python Software Foundation, version 3.9.1, https://www.python.org/downloads/release/python-391/).

**Fig. 11:**
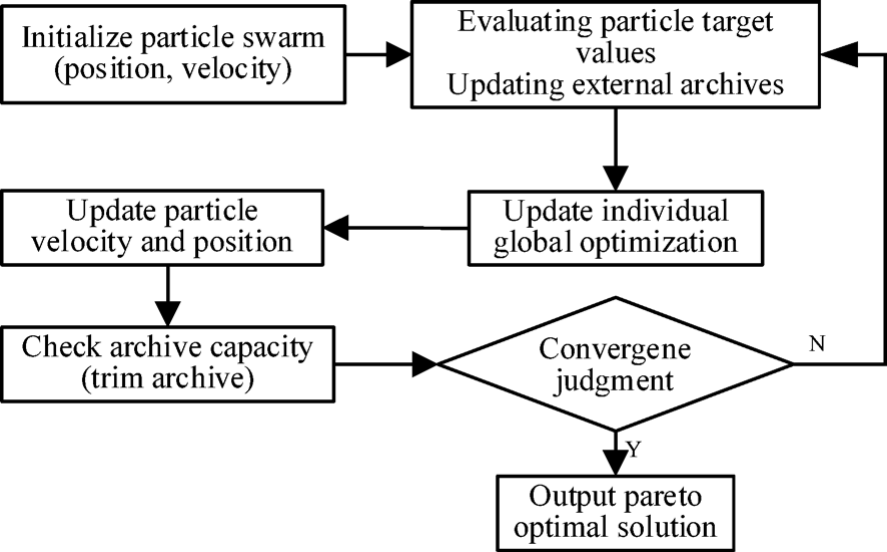
Flowchart of optimization of MOPSO algorithm.

In this paper, we use the MOPSO^30^, which is a stochastic search algorithm based on swarm intelligence specifically designed for solving multi-objective optimization problems. In MOPSO, each combination of variables (X_1_, X_2_, ., X_n_ ) of the objective function is considered as a particle representing a potential solution in the search space. Each particle dynamically updates its speed and position by tracking its individual historical optimal solution and the population global optimal solution. Through iterative optimization, the particle swarm gradually approaches the Pareto front, and finally obtains a set of uniformly distributed and diversified optimal solutions. The MOPSO optimization flow is shown in Fig. 11.

**Fig. 12:**
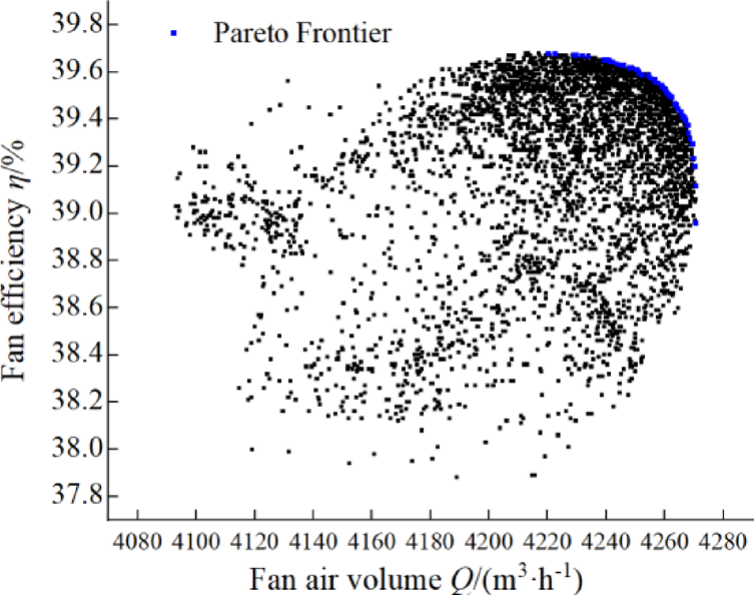
Pareto optimal solution set with iterative scatter plot.

The population size is set to 40 and the maximum number of iterations is 300. The inertia weight is set to 0.7, and the acceleration coefficients are 1.5 and 1.7 respectively. The maximum capacity is set to 100 to store non-dominated solutions generated during the optimization process. Gaussian mutation is adopted, with the mutation probability set to 0.1; the perturbation amplitude of particle positions is 5% of the range of design variables, which serves to break the local convergence of the particle swarm and maintain population diversity. Boundary clamping is implemented via the upper and lower bounds of design variables to ensure all particle positions are within the feasible region. Based on the simulation results, it was found that the Pareto frontier had a negligible effect on the optimization results after increasing to 100 iterations. Therefore, after 100 iterations of computational solving, the Pareto optimal solution set of multi-objective optimization and the iteration scatter plot are obtained, as shown in Fig. 12. With the increase of fan airflow, the speed of fan efficiency reduction increases gradually. In the fan air volume and fan efficiency has the same weight, after comprehensive consideration in the more gentle region to choose the optimal blade parameter combinations are: *β*_1_ = 75.73°, *β*_2_ = 144.67°, *L*_1_ = 41.71 mm, *L*_2_ = 37.29 mm, *γ*_1_ = 14.43°. With this set of parameters to establish a finite element model, in the same impeller speed, respectively, the original fan and the optimized fan for numerical calculation and fan aerodynamic performance experiments, the experimental results are shown in Table 3. The experimental results of the optimized fan were compared with those of the original fan, and the fan air volume was increased by 4.88%, while the fan efficiency was increased by 9.00%; the numerical simulation results of the optimized fan were compared with those of the original fan, and the air volume was increased by 4.03%, while the fan efficiency was increased by 8.38%.

**Table 3:** Comparison of results between experimental and simulated values.

Fan	Results	Q (m^3^/h)	η (%)
Optimized fan	Experimental value	4105.47	36.94
	Analog value	4197.39	38.52
Original fan	Experimental value	3929.28	33.89
	Analog value	4034.77	35.54
Experimental value improvement percentage (%)		4.48	9.00
Analog value improvement percentage (%)		4.03	8.38

## Flow field results analysis

### Characterization of flow field distribution inside the fan

**Fig. 13:**
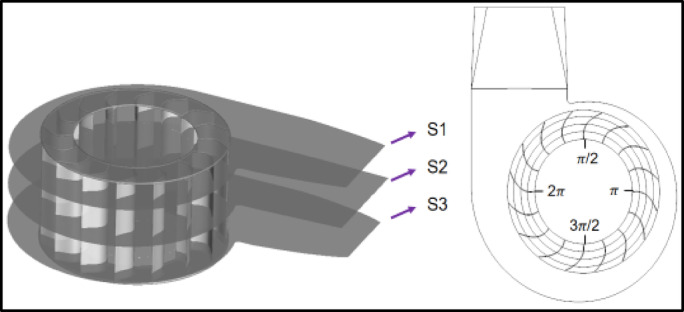
Observation surface of fan flow field.

Based on the numerical simulation results obtained before and after optimization, the maximum air volume and the highest efficiency group before and after optimization at the rated working speed of 1420 r/min were selected for analysis, and to find out the impact of changing the inlet and outlet installation angle, blade inlet and outlet diameter, and blade center angle. In order to facilitate the analysis of the flow field characteristics of different locations within the fan, along the fan axial distance were taken H = 0.050 m at the cross-section S1 (near the impeller inlet), H = 0.125 m at the cross-section S2 (located in the middle plate of the impeller), H = 0.200 m at the cross-section S3 (away from the impeller inlet), and the impeller clockwise delineation of the four observations π/2, π, 3 π/2, 2 π, as shown in Fig. 13.

**Fig. 14:**
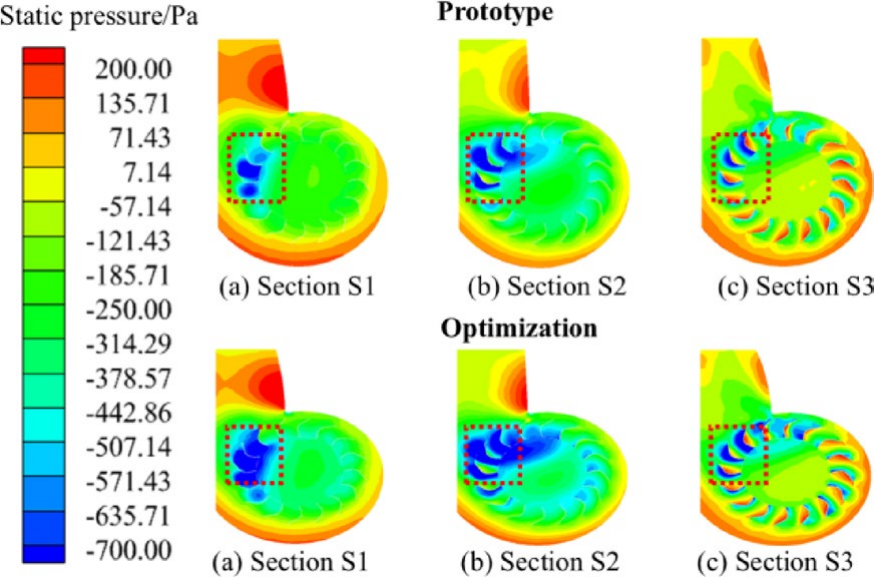
Static pressure distributions in different cross sections of the centrifugal fan.

In order to analyze the flow characteristics of the internal flow field of the centrifugal fan before and after the optimization more deeply, we select the static pressure distributions of the cross-sections *S*1, *S*2 and *S*3 of the fan as a whole, as shown in Fig. 14. As the airflow flowed from the inlet to the outlet of the impeller, the static pressure gradually increased. At the front end of the worm tongue, a localized low-pressure region appears (the boxed portion of the figure). The optimized fan creates a more pronounced low-pressure zone in the inlet area, a phenomenon that is attributed to the optimized design, which creates a stronger centrifugal force in the fan and allows the airflow to flow more smoothly into the impeller ducts. At the same time, the low-pressure zone at the front of the tongue is not only widened, but the pressure is further reduced, allowing the airflow to enter the ducts more fully after flow separation, thus increasing the efficiency of the fan. From a macro point of view, these improvements significantly increase the airflow output of the fan, indicating that the optimized design has a significant effect in improving the performance of the fan.

**Fig. 15:**
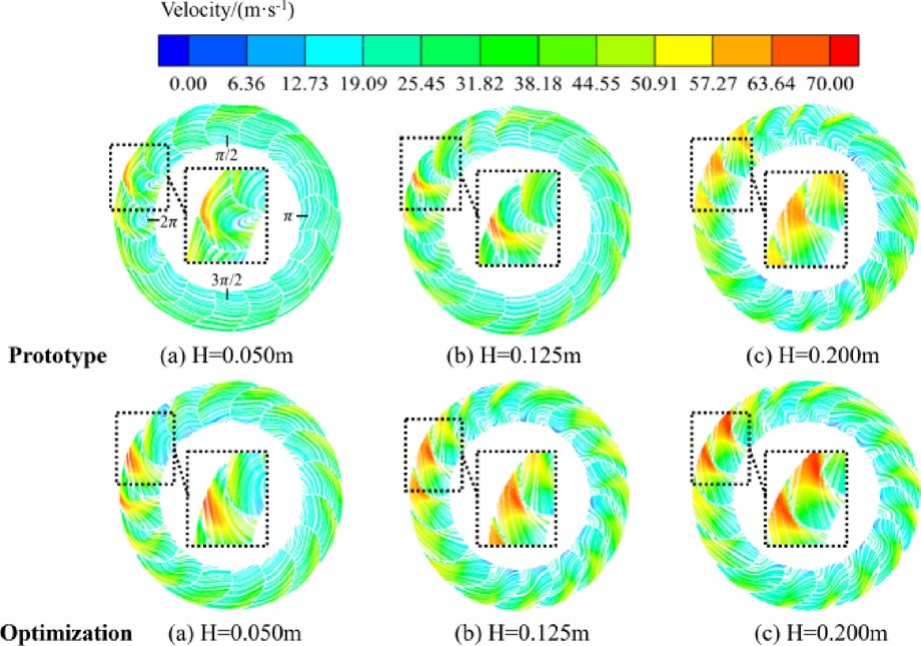
Velocity fields and streamlines distributions in different cross sections of the centrifugal fan.

Figure 15 shows a comparison of the velocity streamlines of the inter-impeller flow paths at different height sections of the centrifugal fan before and after optimization. From the figure, it can be seen that the degree of flow separation of airflow varies in different cross-sections, which in the front of the worm tongue to form a high-speed region, and in the impeller air inlet at the lower speed, especially at the air inlet. Therefore, it can be concluded that the main trend of airflow velocity is gradually increasing from the impeller inlet to the outlet. In the fan’s inter-impeller flow path is very easy to form a vortex of varying sizes, which will lead to a certain degree of flow separation and reduce the energy efficiency of the fan. Before and after the optimization of the fan flow field distribution in the front of the tongue as well as the inlet and outlet and other parts show significant differences. Before optimization, under the rated condition of the fan, the airflow distribution through the worm shell and impeller was more uneven, some flow lines showed backflow regions, and near the blade exit, the flow was more turbulent, which directly affects the fan’s air volume and efficiency^31^. In this case, the energy loss of the airflow is large, resulting in unsatisfactory fan efficiency. The optimized fan has significantly improved the flow field inside the impeller. The optimized design adjusts the mounting angle of the blades and the curvature of the blades so that the airflow can flow more smoothly on the surface of the blades. The distribution of the flow field is more uniform, especially in the impeller outlet area, the phenomenon of backflow is significantly reduced, and the energy loss of the airflow is effectively suppressed. This change effectively reduces the aerodynamic resistance, improves the working efficiency of the fan, and significantly increases the air volume.

**Fig. 16:**
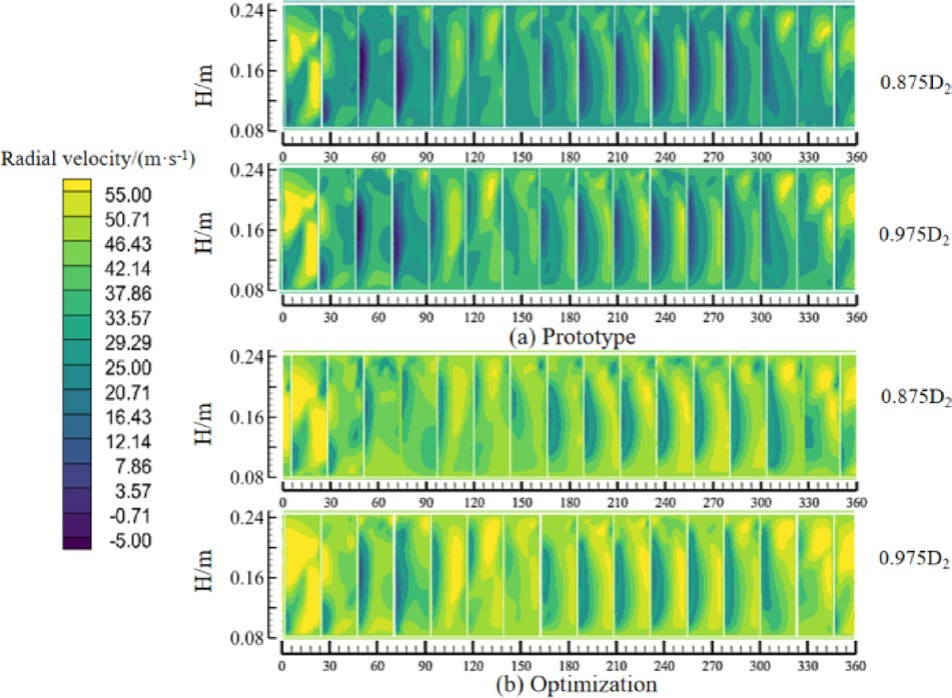
Turbulence energy in the centrifugal fan and its tongue.

Turbulent kinetic energy is mainly used to characterize the turbulence intensity and flow stability, Fig. 16 shows the distribution of turbulent kinetic energy in 50% of the impeller width section and the worm tongue area. Compared with the original turbulence pulsation inside the fan, the optimized fan has effectively suppressed the strong turbulence energy phenomenon in the fan inlet and outlet positions and the worm tongue area; the pressure pulsation on the worm tongue surface caused by turbulence pulsation is significantly reduced, which indicates that the optimized centrifugal fan has a more stable flow inside the fan, and the reduction of turbulence kinetic energy also indicates that the optimized fan has less internal energy loss, and the mechanical efficiency of the fan is also improved. The mechanical efficiency of the fan is also improved. The blade profile designed by the third-order Bezier curve improves the stability of the internal flow state of the fan, effectively reduces the secondary flow and airflow impact in the cross section of the worm tongue, and reduces the energy loss of the fan.

**Fig. 17:**
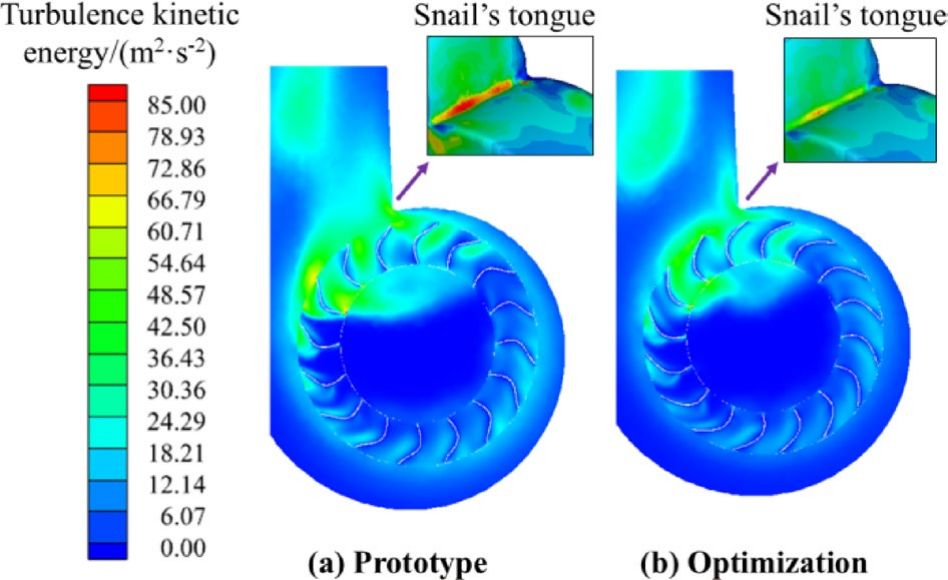
Comparison of velocities in the impeller outlet section.

The radial velocity distribution of the impeller outlet cross-section is shown in Fig. 17, and the surface expansion diagrams of 0.875 and 0.975 times of the impeller outer diameter *D*_2_ are made along the radial distance. From the radial velocity cloud diagram of the impeller outlet before and after optimization, it can be seen that the radial velocity is asymmetrically distributed along the circumference, and the overall velocity of the impeller channel before optimization is low, and the velocity in some areas of the phenomenon is negative, which is a certain obstacle to the internal flow of the fan. After optimization, the impeller outlet radial velocity is uniformly improved, which is macroscopically manifested in the increase of centrifugal fan air volume and the improvement of impeller airflow backflow phenomenon.

## Shaking green test

### Test sample

**Fig. 18:**
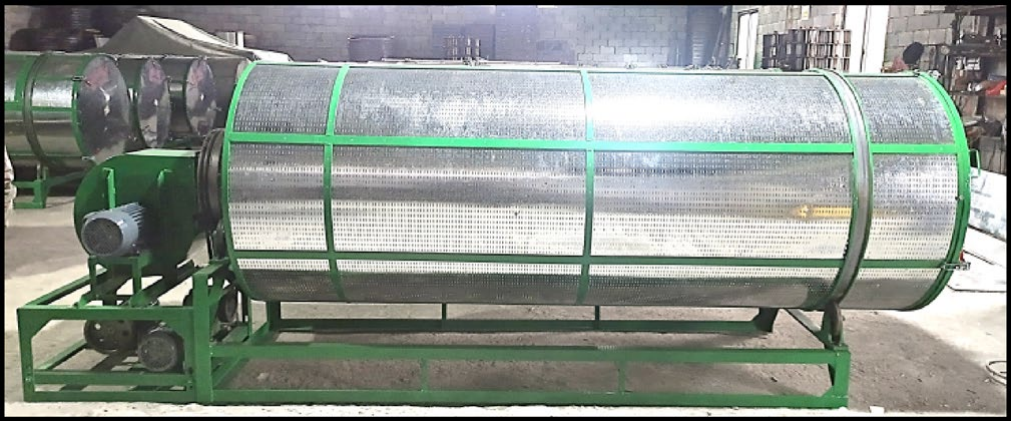
Oolong tea shaking machine.

In order to systematically assess the quality enhancement effect of centrifugal fan optimization on oolong tea shaking process, Wuyi Rock Tea was selected to carry out a two-group control study. As shown in Fig. 18, the tea green withered by sunlight is sent into the shaking machine for shaking, and the continuous friction between the leaf blade and the rotary leaf roller causes the leaf edge cells to break, accelerating the enzymatic oxidation of tea polyphenols and the release of aromatic substances, forming the iconic “green leaves with red edges”, and the centrifugal fan drives the hot air circulation to promote the migration of water gradient. Based on this process characteristics, this test selected the shaking process at different times of the stalk, leaf parts of the moisture content and the sensory evaluation of the sampled tea samples as the core evaluation index, through the comparison of the two sets of data before and after the optimization, to verify the centrifugal fan to improve the effect of the shaking process.

**Fig. 19:**
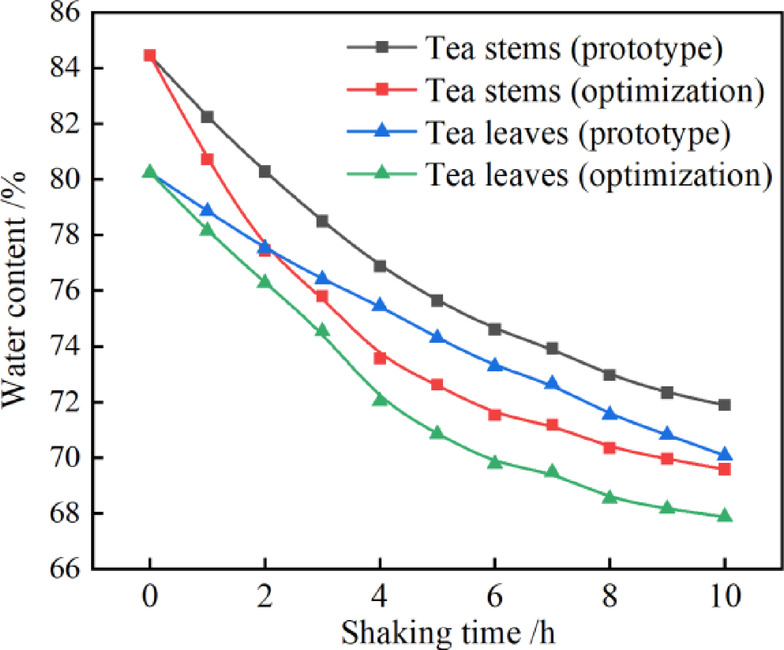
Rock tea stems and leaves: moisture content of tea change after shaking optimization.

According to the “Determination of Tea Moisture” in the national standard GB/T 8303 − 2013 “Tea—Preparation of ground sample and determination of dry matter content”, the formula for calculating the moisture content of tea is shown in Eq. (4), calculate the moisture content of tea processing

4 $$\:{\omega\:}_{i}=\frac{{m}_{i}-{m}_{0}}{{m}_{0}}\times\:100\%$$

where $\:{\omega\:}_{i}$ is the moisture content of tea leaves at time *i*, $\:{m}_{i}$ is the mass of tea leaves at time *i*, and $\:{m}_{0}$ is the mass of 1 kg of tea leaves after drying. The moisture content of tea stalks and leaves during processing is shown in Fig. 19. Each batch of tea samples is set to 1 kg (consistent with the actual production scale of oolong tea processing), with 3 test repetitions to ensure reproducibility. Fresh tea leaf sampling is strictly in accordance with GB/T 8302 − 2013 Tea - Sampling (Clause 5.1) — representative samples are randomly collected from the upper, middle and lower layers of the withered fresh tea leaf pile using the diagonal quartering method to avoid sampling bias. For moisture content determination, each sample is further divided into 3 parallel subsamples to reduce random errors.

As can be seen from Fig. 19, under the condition of 10 h of shaking, the moisture content of leaf stalk and leaf surface of rock tea decreased greatly in the early stage, and the decrease was small in the later stage, and the moisture content of leaf stalk was still higher than that of leaf blade. From the comparison, it can be seen that after shaking with the optimized equipment, the reduction in moisture content of the rock tea leaf stalks and blades was less than that with the original equipment, and the reduction rate was faster. In the appropriate shaking time of 8 h, the optimized equipment compared with the prototype machine after shaking the level of the leaf stalk part of the water level was increased by 3.57%, the level of the leaf blade water level was increased by 4.21%. The same time the optimized equipment tea water effect is more significant, this is because the optimized blade profile is more in line with the air flow, impeller work effect is significant, improve the centrifugal fan air volume, to achieve a better shaking effect.

### Tea sensory evaluation scores

**Fig. 20:**
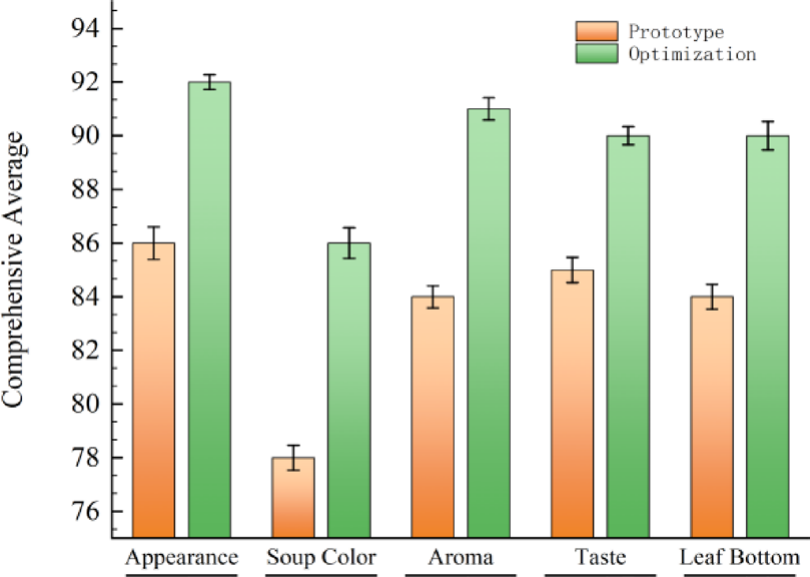
Comparison of sensory evaluation of tea under shaking condition before and after optimization. *(Composite Score = Appearance Characteristics Score × 20% + Soup Color Score × 30% + Aroma Score × 30% + Taste Score × 10% + Leaf Bottom Characteristics Score × 10%).

In accordance with GB/T 23,776 − 2018 “Methodology for Sensory Evaluation of Tea”, quantitative sensory quality evaluation was conducted on rock tea samples processed by original and optimized equipment, focusing on five factors: shape, liquor color, aroma, taste, and infused leaf. The 95% confidence intervals (CI) and adjusted sensory characteristics of each indicator before and after optimization are shown in Table 4. From the sensory score comparison in Fig. 20, it can be seen that all sensory indicator scores after optimization were significantly higher than those before optimization, and all P-values were < 0.01, indicating extremely significant differences. Experimental data showed that the comprehensive sensory quality index of tea in the optimized group increased from 82.60 (control group) to 89.40, meeting the premium grade threshold (≥ 87 points) specified in the “Oolong Tea” section of GB/T 30,766 − 2014 “Classification of tea”. The results confirm that the optimized equipment improves the sensory evaluation score of processed tea by one grade, significantly enhancing the shaking effect of the oolong tea shaking machine.

**Table 4:** Oolong tea sensory optimization: CI and evaluation comparison.

Sensory indicator	Prototype		Optimization	
	average score	95% CI	Average score	95% CI
Appearance	86	84.33–87.67	92	91.23–92.77
Soup color	78	77.13–78.07	86	85.13–86.27
Aroma	84	82.85–85.15	91	89.85–92.15
Taste	85	83.70–86.30	90	89.07–90.93
Leaf bottom	84	82.71–85.29	90	88.52–91.48
Composite score	82.60		89.40	
Adjusted sensory Evaluation	Appearance: Relatively heavy and sturdy, with variety or regional characteristics, lustrous, relatively uniform, fairly good cleanliness		Appearance: Heavy and tight, with obvious variety or regional characteristics, oily luster, uniform, good cleanliness	
	Soup Color: Depends on processing technology, relatively bright		Soup Color: Depends on processing technology, ranging from honey yellow to orange red, but required to be clear and bright	
	Aroma: Fairly obvious variety or regional characteristics, with floral or fruity aroma, but slightly less rich and pure		Aroma: Obvious variety or regional characteristics, rich floral or fruity aroma, elegant and pure	
	Taste: Fairly mellow and refreshing, fairly mellow and smooth		Taste: Thick, mellow and sweet or mellow and smooth	
	Leaf Bottom: Relatively soft and bright leaves, fairly well made during the oxidation process		Leaf Bottom: Thick, soft and bright leaves, well made during the oxidation process	

## Conclusion

In this paper, the impeller of centrifugal fan used in the shaking green barrel was modeled with parameters, and the design variables of the centrifugal fan blade were optimized by MOPSO. Finally, it was determined that when the inlet mounting angle *β*_1_ = 75.73 , outlet mounting angle *β*_2_ = 144.67 , blade inlet diameter *L*_1_ = 41.71 mm, blade outlet diameter *L*_2_ = 37.29 mm, blade center angle *γ*_1_ = 14.43°. The optimum values of fan air volume and fan efficiency are 4197.39 m^3^/h and 38.52%. The optimized fan increased the airflow by 4.48% and efficiency by 9.00% compared to the original fan. The comparison between numerical simulation and experimental results verifies the effectiveness of the optimization scheme.

Through GSA, it was determined that the blade center angle $\:{\gamma\:}_{1}$ had the greatest influence on the air volume of the centrifugal fan, while the blade outlet angle *β*_2_ and the blade inlet angle *β*_1_ had an important influence on the efficiency of the wind turbine. The key design variables affecting the performance of the fan were identified through the global sensitivity analysis, which provided a theoretical basis for the subsequent optimization. Further analysis shows that optimizing the blade inlet and outlet angles and diameters not only enhances the wind capacity of the fan, but also effectively improves its operating efficiency.

Through the optimized design, the internal flow field of the fan is significantly improved, especially in the front of the worm tongue and the inlet and outlet areas. The optimized flow field of the fan is more uniform, the phenomenon of backflow is reduced, the turbulent kinetic energy is effectively suppressed, the energy loss of the airflow is reduced, and the overall efficiency is improved. These improvements in the flow field directly promote the improvement of fan performance.

In practical application, the optimized centrifugal fan effectively enhanced the performance of the oolong tea shaking machine under actual working conditions. For Wuyi Rock Tea, the moisture loss of leaf stalks and blades increased by 3.57 and 4.21% points, respectively, and the average sensory evaluation score of the processed tea increased by 6.8 points, meeting the premium grade standard specified in relevant national standards, By integrating the Kriging surrogate model and MOPSO algorithm, it realizes multi-parameter collaborative optimization of centrifugal fans, improves aerodynamic performance, and provides theoretical and technical support for the intelligent upgrading of tea processing equipment. However, this study is subject to limitations, including a constrained parametric scope, limited diversity in experimental samples, and the simplifying assumptions inherent in the CFD model. Subsequent work can expand the scope of optimization parameters develop exclusive parameter schemes for different oolong tea varieties such as Tieguanyin and Da Hong Pao, conduct long-term industrial verification to promote industrial application.

## Data Availability

All data generated or analyzed during this study are included in this manuscript.

## Abbreviations

CFD: Computational fluid dynamics
CI: Confidence interval
GSA: Global sensitivity analysis
k-ε: K-epsilon model
MOPSO: Multi-objective particle swarm optimization
NSGA-II: Non-dominated sorting genetic algorithm II
OLHS: Optimal latin hypercube sampling
PRESTO!: Pressure staggering option
R^2^: Coefficient of determination
RMSE: Root mean square error
SVR: Support vector regression

## List of symbols

$\:{L}_{1}$: Blade inlet diameter
$\:{L}_{2}$: Blade outlet diameter
$\:Z$: Number of blades
$\:{D}_{1}$: Impeller outer diameter
$\:{D}_{2}$: Impeller inner diameter
$$\:Q$$: Air volume
$\:\overline{{u}_{i}}$: The time-averaged velocity component in the i-th direction
$\:\overline{{u}_{j}}$: The time-averaged velocity component in the j-th direction
$\:{x}_{i}$: The i -th spatial coordinate
$\:{x}_{j}$: The j-th spatial coordinate
$$\:P$$: Pressure
$\:{d}_{i}$: The Euclidean distance vector between any two sample points in the design space
$\:{\theta\:}_{i}$: The anisotropic scaling factor for design variable
$\:{m}_{0}$: The mass of 1 kg tea leaves after drying
$\:{m}_{i}$: The mass of tea leaves at time i

## Greek symbols

$\:{\beta}_{1}$: Inlet mounting angle
$\:{\beta}_{2}$: Outlet mounting angle
$$\:\rho\:$$: Density
$\:{\gamma}_{i}$: Blade center angle
$$\:v$$: Viscosity coefficient
$$\:\eta\:$$: Efficiency
$\:{\omega}_{i}$: Tea leaf moisture content at time
